# Commercial Enterprise vs. Paternalism

**Published:** 1900-03

**Authors:** 


### COMMERCIAL ENTERPRISE *vs.* PATERNALISM.

THE battle is on between those who are commercially engaged in the preparation of biological products and our government, which, while ostensibly engaged in scientific investigation, is, on the other hand, filling the role of a paternal government contrary to the teachings and foundation-stones of our country. The free access to these products is destined to ultimately become as much a sore on the body politic as the shameful seed scandal of our parental government. The open door of correspondence between those directing the Department of Agriculture and the commercial makers and venders of their products has assumed strong proportions, and our Congressional representatives at the nation's capital find a burning question to solve. Much can be said on both sides of this problem, and in these days of sudden changes there seems a much stronger disposition than ever to have our national government regulate our railways, telegraph and telephone lines as well as our postal service. On the other hand, the freedom of trade to be afforded to every industry by our parental government is not given in this direction when the utmost latitude of generosity and paternalism prevails in the free distribution of these products, prepared, etc., at governmental expense. Another great danger of much importance to scientific advancement is the furnishing of these preparations by both parties to laymen, thus endangering their proper and wise use, not to speak of the danger as well as nearly valueless deductions drawn from the reports received in return. Such work as this should ever remain in scientific hands, where all its dangers, uncertainties, etc., are fully recognized and intelligently considered.

